# PmrB Y358N, E123D amino acid substitutions are not associated with colistin resistance but with phylogeny in *Escherichia coli*

**DOI:** 10.1128/spectrum.00532-24

**Published:** 2024-08-20

**Authors:** Alyssa Butters, Juan Jovel, Sheryl Gow, Karen Liljebjelke, Cheryl Waldner, Sylvia L. Checkley

**Affiliations:** 1 Faculty of Veterinary Medicine, University of Calgary, Calgary, Alberta, Canada; 2 AMR—One Health Consortium, Calgary, Alberta, Canada; 3 Canadian Integrated Program for Antimicrobial Resistance Surveillance/FoodNet, Public Health Agency of Canada, Ottawa, Ontario, Canada; 4 Department of Large Animal Clinical Sciences, Western College of Veterinary Medicine, University of Saskatchewan, Saskatoon, Saskatchewan, Canada; Centre de Biologie Integrative, Toulouse, France

**Keywords:** *Escherichia coli*, antimicrobial resistance, colistin, PmrB, molecular epidemiology

## Abstract

Colistin resistance in *Escherichia coli* is of public health significance for its use to treat multidrug-resistant Gram-negative infections. Amino acid variations in PmrB have been implicated in colistin resistance in *E. coli*. In this cross-sectional study, 288 generic *E. coli* isolates from surveillance of broiler chicken and feedlot cattle feces, retail meat, wastewater, and well water were whole-genome sequenced. Phylogroup designation and screening for two amino acid substitutions in PmrB putatively linked to colistin resistance (Y358N, E123D) were performed *in silico*. Three additional data sets of publicly available *E. coli* assemblies were similarly scrutinized: (i) *E. coli* isolates from studies identifying the Y358N or E123D substitutions, (ii) colistin-susceptible *E. coli* isolates reported in the literature, and (iii) a random sampling of 14,700 *E. coli* assemblies available in the National Center for Biotechnology Information public database. Within all data sets, ≥95% of phylogroup B1 and C isolates have the PmrB Y358N variation. The PmrB E123D amino acid substitution was only identified in phylogroup B2 isolates, of which 94%–100% demonstrate the substitution. Both PmrB amino acid variations were infrequent in other phylogroups. Among published colistin susceptible isolates, colistin minimum inhibitory concentrations (MICs) were not higher in isolates bearing the E123D and Y358N amino acid variations than in isolates without these PmrB substitutions. The E123D and Y358N PmrB amino acid substitutions in *E. coli* appear strongly associated with phylogroup. The previously observed associations between Y358N and E123D amino acid substitutions in PmrB and colistin resistance in *E. coli* may be spurious.

**IMPORTANCE:**

Colistin is a critical last-resort treatment for extensively drug-resistant Gram-negative infections in humans. Therefore, accurate identification of the genetic mechanisms of resistance to this antimicrobial is crucial to effectively monitor and mitigate the spread of resistance. Examining over 16,000 whole-genome sequenced *Escherichia coli* isolates, this study identifies that PmrB E123D and Y358N amino acid substitutions previously associated with colistin resistance in *E. coli* are strongly associated with phylogroup and are alone not sufficient to confer a colistin-resistant phenotype. This is a critical clarification, as both substitutions are identified as putative mechanisms of colistin resistance in many publications and a common bioinformatic tool. Given the potential spurious nature of initial associations of these substitutions with colistin resistance, this study's findings emphasize the importance of appropriate experimental design and consideration of relevant biological factors such as phylogroup when ascribing causal mechanisms of resistance to chromosomal variations.

## INTRODUCTION

Gram-negative infections represent a substantial burden to human health. With multidrug-resistant Gram-negative infections increasing and few new antimicrobials available to treat them, pharmaceutical therapy for multidrug-resistant infections has increasingly relied on last-resort antibiotics such as colistin, especially to treat infections carbapenem-resistant organisms (1, 2).

Although colistin was first discovered in 1947 and developed for use in the 1950s, its nephrotoxicity and neurotoxicity limited its use in humans (2, 3). However, it was used extensively in food animal production in many areas worldwide to control colibacillosis and diarrhea in animals (2, 4). In response to the emerging need to use colistin as a salvage therapy, restrictions on the use of colistin in food animals have since been introduced in many jurisdictions (4). Surveillance for colistin resistance in human, animal, and environmental contexts and understanding the mechanisms of resistance will be crucial to tracing the spread of colistin resistance (5) and preserving the future utility of colistin as a last-resort antimicrobial chemotherapy. As a common commensal and environmental organism but also a frequent and important opportunistic Gram-negative pathogen (4, 6), *Escherichia coli* is of particular interest in the study of colistin resistance.

Colistin (polymyxin E) exerts its effect on *E. coli* and other Gram-negative bacteria by binding to lipopolysaccharide (LPS) in the outer cell membrane, causing increased membrane permeability and, ultimately, lytic cell death (2). Cellular alterations resulting in colistin resistance typically consist of post-translational modifications adding cationic molecules such as 4-amino-4-deoxy-L-arabinose (L-Ara4N) and phosphoethanolamine (PEtN) to the lipopolysaccharide (LPS) (3, 4), impeding the binding of cationic polymyxin antibiotics (3). PmrA/PmrB is a two-component system crucial to the synthesis of PEtN and the regulation of LPS modification (1–3).

Acquired resistance to colistin, mediated by plasmids bearing *mcr* genes encoding phosphoethanolamine (PEtN) transferases (2, 7), has been well-established. However, colistin resistance mechanisms not mediated by *mcr* genes are less understood. Quesada et al. (8) and Cannatelli et al. (9) were the first to report colistin-resistant *E. coli* that demonstrated mutations in the PmrB sensor kinase and speculated these mutations may be associated with the resistant phenotype. Many others have also identified mutations in PmrB, including Y358N and E123D amino acid substitutions, in colistin-resistant *E. coli* isolates with or without concurrent *mcr*-mediated mechanisms of resistance (7–18). The Y358N and E123D amino acid variations are included in the AMRFinderPlus bioinformatic tool (19) as markers of putative colistin resistance. However, the correlation of these point mutations with colistin has not been absolute, as the E123D and Y358N amino acid substitutions are reported in colistin-susceptible *E. coli* (20, 21). Despite this, these associations are still identified and frequently noted in the literature as conferring colistin resistance (12–16).

The aim of this study was to assess relationships between phylogroup designation, phenotypic colistin susceptibility, and the presence of PmrB Y358N and E123D amino acid substitutions in whole genome sequences of a large set of generic *E. coli* isolates collected from routine surveillance projects. Three datasets of publicly available *E. coli* genomes were also scrutinized for associations between the Y358N/E123D PmrB amino acid substitutions, colistin susceptibility (when available), and phylogroup: (i) isolates from published studies identifying the Y358N or E123D PmrB amino acid variations, (ii) isolates reported in the literature to be non-resistant to colistin by broth dilution, and (iii) *E. coli* assemblies sampled randomly from the National Center for Biotechnology Information (NCBI) public database.

## RESULTS

**Fig 1:**
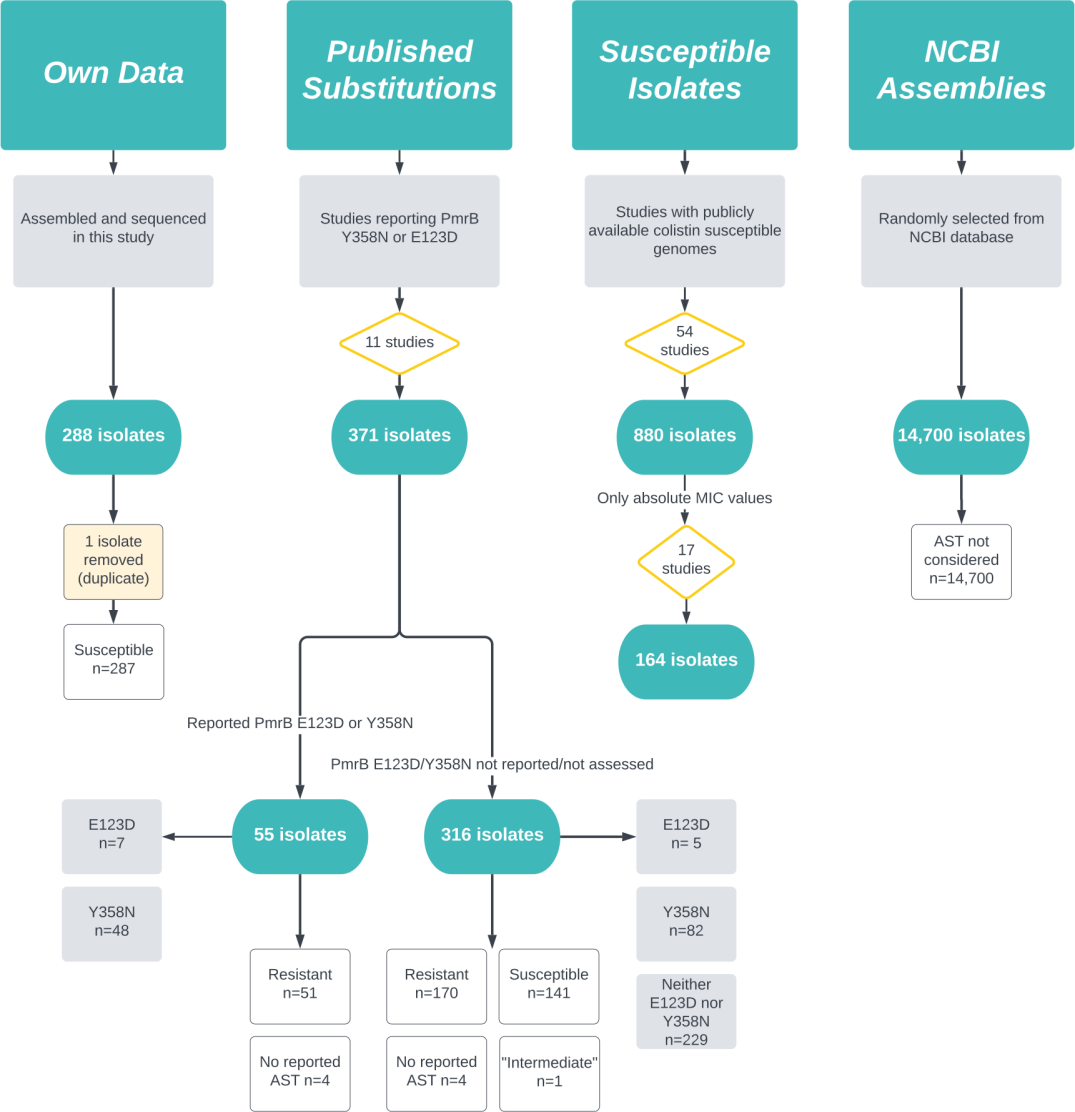
Datasets examined in this study.

Whole-genome assemblies of over 16,000 *E. coli* isolates were assessed for phylogroup and the presence of the PmrB E123D and Y358N amino acid variations (Materials and Methods, Fig. 1). Phenotypic colistin susceptibility was available for 1,504 of these isolates.

**Fig 2:**
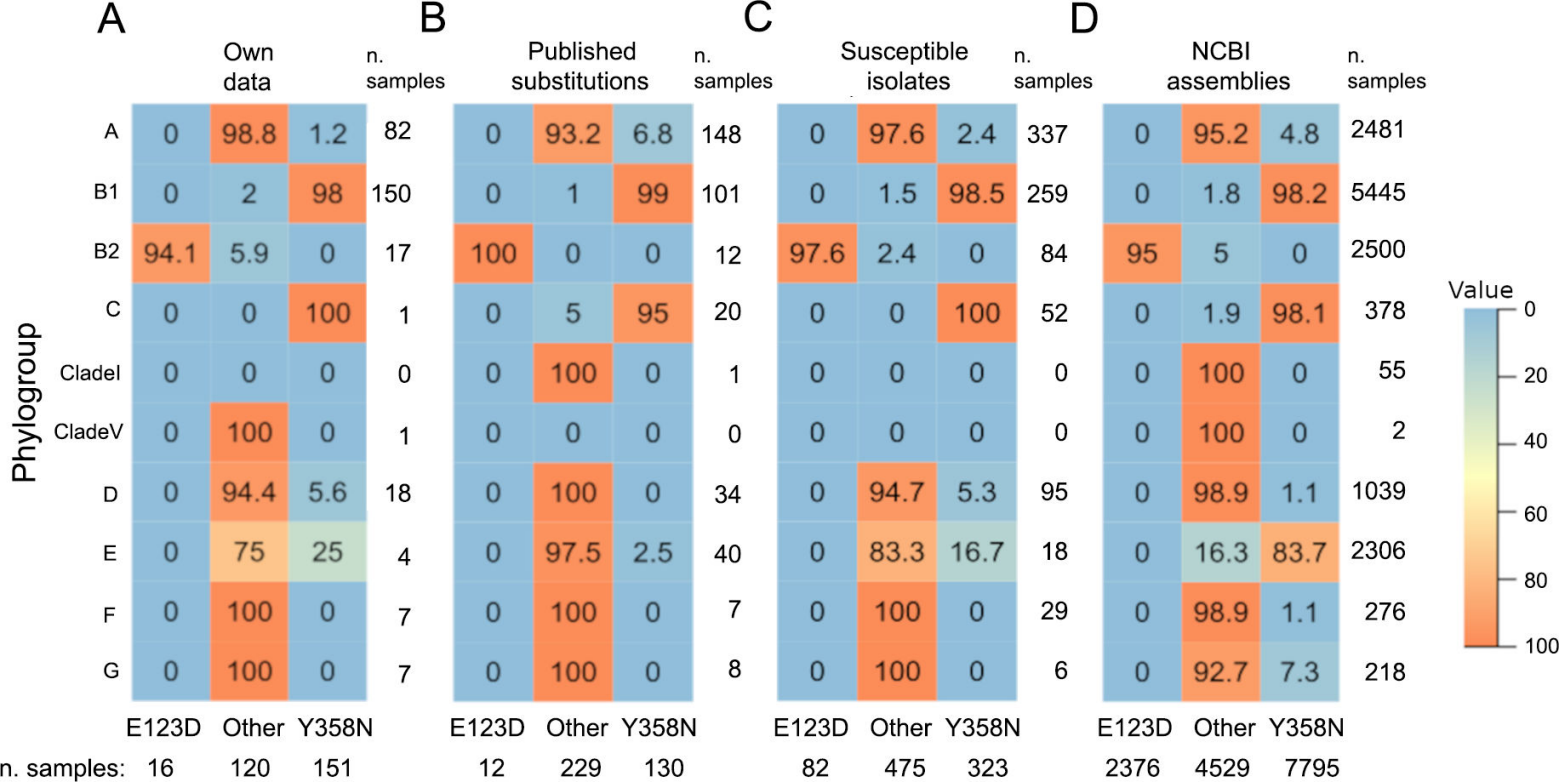
Phylogroup distributions of *E. coli* isolates demonstrating the presence or absence of PmrB E123D and Y358N amino acid substitutions among (A) 287 *E. coli* isolates from retail beef, retail chicken, well water, wastewater, and broiler chicken and feedlot cattle feces sequenced for this study (*Own Data*), (**B**) *E. coli* genomes from publications identifying PmrB Y358N or E123D amino acid variations (*Published Substitutions*), (**C**) colistin-susceptible *E. coli* isolates reported in the literature (*Susceptible Isolates*), and (D) randomly selected *E. coli* assemblies downloaded from the National Center for Biotechnology Information database (*NCBI Assemblies*). Cell values are percentages of all isolates in each phylogroup that demonstrate the PmrB E123D amino acid variation, other variation, or PmrB Y358N variation (row-wise).

Of the 288 *E. coli* isolates sequenced from broiler chicken fecal, feedlot fecal, retail beef and chicken meat, wastewater, and well water (data set hereafter called *Own Data*), one isolate was found to be a duplicate and was therefore excluded. Of the remaining 287 isolates, 151 (52.6%) bore the PmrB Y358N amino acid substitution associated with putative colistin resistance, dominated by isolates from phylogroup B1 (97.4%, Table 1). Almost all B1 isolates carried the Y358N variation (98.0%, Fig. 2). No other phylogroup contained more than one isolate bearing the Y358N variation, however, the Y358N-bearing phylogroup C isolate was the only phylogroup C isolate in the data set. All isolates demonstrating the PmrB E123D amino acid variation belonged to phylogroup B2, whereas only 5.8% of phylogroup B2 isolates (*n* = 1) did not bear either the E123D or the Y358N substitution.

**TABLE 1:** Phylogroup, PmrB amino acid variations, and phenotypic colistin susceptibility

Phylogroups	PmrB amino acid substitution		
	E123D	Neither Y358N nor E123D	Y358N
*Own Data^a^*			
A	0	81	1
B1	0	3	147
B2	16	1	0
C	0	0	1
Clade I	0	0	0
Clade V	0	1	0
D	0	17	1
E	0	3	1
F	0	7	0
G	0	7	0
TOTAL	16	120	151
*Published Substitutions—all isolates^b^*			
A	0	138	10
B1	0	1	100
B2	12	0	0
C	0	1	19
Clade I	0	1	0
Clade V	0	0	0
D	0	34	0
E	0	39	1
F	0	7	0
G	0	8	0
TOTAL	12	229	130
*Susceptible Isolates^c^*			
A	0	329	8
B1	0	4	255
B2	82	2	0
C	0	0	52
Clade I	0	0	0
Clade V	0	0	0
D	0	90	5
E	0	15	3
F	0	29	0
G	0	6	0
TOTAL	82	475	323
*NCBI Assemblies^d^*			
A	0	2,363	118
B1	0	98	5,347
B2	2,376	124	0
C	0	7	371
Clade I	0	55	0
Clade V	0	2	0
D	0	1,028	11
E	0	377	1,929
F	0	273	3
G	0	202	16
TOTAL	2,376	4,529	7,795

^*a*^ 287 uniquely sequenced *E. coli* isolates from well water, wastewater, broiler chicken and feedlot cattle feces, retail beef and retail chicken meat*.*

^*b*^ *E. coli* genomes from publications identifying PmrB Y358N or E123D amino acid variations.

^*c*^ Colistin-susceptible *E. coli* isolates reported in the literature.

^*d*^ Randomly selected *E. coli* assemblies downloaded from the National Center for Biotechnology Information database.

None of the *E. coli* isolates from the *Own Data* data set demonstrated growth on Mueller-Hinton agar containing 2 mg/L colistin sulfate, the Clinical and Laboratory Standards Institute (CLSI) and European Committee on Antimicrobial Susceptibility Testing (EUCAST) susceptibility breakpoint concentration (22, 23).

Given the observed phylogroup patterns noted among isolates with the PmrB E123D and Y358N substitutions in the *Own Data* data set, the phylogroup designations of published isolates reported to bear the E123D or Y358N PmrB substitutions were explored. The literature was surveyed for studies reporting one or more *E. coli* isolates with either PmrB Y358N or E123D amino acid substitutions and having publicly available genomes so the phylogroup designation of the substitution-bearing isolates could be determined. Eleven studies met these criteria (4, 12, 15, 16, 24–30) (hereafter referred to as the *Published Substitutions* data set). A total of 55 isolates within the 11 studies demonstrated one of the two variations of interest (Fig. 1). Although phenotypic colistin susceptibility was not reported for four isolates (27), the remaining 51 isolates were reported to be phenotypically resistant to colistin. Forty-eight isolates were reported to bear the Y358N substitution, and seven isolates were reported with the E123D substitution. As seen in the *Own Data* data set, all E123D-bearing isolates reported in these studies were found to belong to phylogroup B2, and a majority of isolates demonstrating PmrB Y358N belonged to phylogroup B1 (83%). All phylogroup C isolates (*n* = 2) bore the Y358N variation.

However, additional *E. coli* genomes (*n* = 316) from the same 11 studies in the *Published Substitutions* data set were also publicly available. These isolates were not assessed for the PmrB substitutions in the studies or were not reported to demonstrate the 2 variations of interest. These additional isolates were downloaded and assayed for phylogroup and the PmrB E123D and Y358N amino acid substitutions (Fig. 1). Among these 316 isolates, 170 were reported to be phenotypically resistant to colistin (53.8%), one was identified as “intermediate” [no minimum inhibitory concentration (MIC) given], and 141 (44.6%) were reportedly phenotypically susceptible. Phenotypic colistin susceptibility was not reported for four isolates (1.3%).

**Fig 3:**
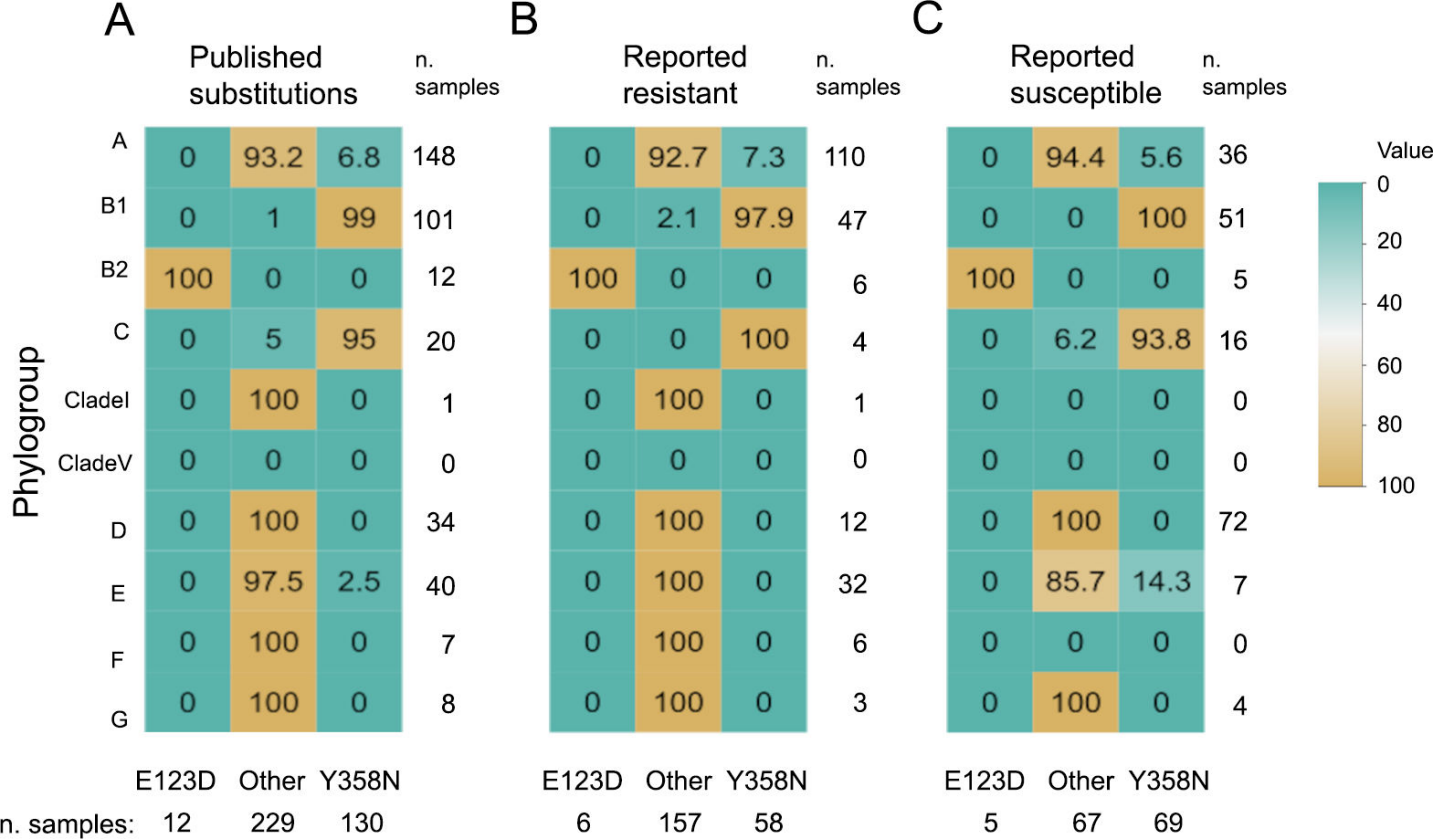
Phylogroup distribution among *E. coli* isolates in 11 published studies reporting one or more isolates with PmrB Y358N and E123D amino acid substitutions. (A) All publicly available *E. coli* isolates in the 11 studies, (B) *E. coli* isolates within the 11 studies that are reportedly phenotypically resistant to colistin, and (C) *E. coli* isolates within the 11 studies that are reportedly phenotypically susceptible to colistin. No phenotypic antimicrobial susceptibility was provided for eight isolates (1 study; 3 B1 isolates, and 1 isolate in each of phylogroups B2, A, E, F, and G). One isolate from phylogroup A was reported as “Intermediate” colistin susceptibility. Cell values are percentages of all isolates in each phylogroup that demonstrate the PmrB E123D amino acid variation, other variation, or PmrB Y358N variation (row-wise).

Considering all 371 publicly available *E. coli* genomes in the *Published Substitutions* data set (55 isolates with reported PmrB E123D/Y358N, 316 without), irrespective of reported phenotypic colistin susceptibility, isolates from phylogroups B1 and C represented 91.5% of the isolates demonstrating the Y358N amino acid variation. In this full data set, all isolates with the E123D variation belonged to phylogroup B2, and conversely, all B2 isolates bore the E123D variation (Fig. 2). Among B1 isolates, 99% demonstrated the Y358N amino acid substitution (*n* = 100), as did 95% of phylogroup C isolates (*n* = 19). Isolates bearing neither the E123D nor the Y358N amino acid substitution (*n* = 229) most frequently belonged to the remaining phylogroups (A, D, E, F, G, Clade I; 99.1%, *n* = 227). When resistant and susceptible isolates in this data set were considered separately, similar trends were seen where 97.9% (colistin-resistant) to 100% (colistin-susceptible) of phylogroup B1 isolates and 93.8% (colistin-susceptible) to 100% (colistin-resistant) of phylogroup C isolates bear the Y358N amino acid substitution (Fig. 3).

As one of the first reports of Y358N and E123D amino acid substitutions in colistin-resistant isolates (12) involved comparison of colistin-resistant isolates to *E. coli* K-12 MG 1655, an assembly of this lab standard was downloaded and confirmed to belong to phylogroup A. Neither the Y358N nor E123D PmrB amino acid substitutions were found in the K-12 assembly.

The observed presence of the Y358N and E123D amino acid substitutions putatively linked to colistin resistance in phenotypically colistin susceptible isolates within the *Own Data* and *Published Substitutions* data sets prompted further exploration of the association between colistin resistance and the PmrB variations of interest. The literature was further searched for additional colistin-susceptible isolates with publicly available whole-genome assemblies. This search yielded 961 non-duplicate studies, of which 54 met the criteria of reporting one or more colistin-susceptible isolates (as determined by broth dilution) with publicly available assemblies or sequence-read archives. Within the 54 studies, 880 susceptible isolates were identified (data set hereafter called *Susceptible Isolates*). Isolates (*n* = 26) from one study (26) in the *Published Substitutions* data set were also included in the *Susceptible Isolates* data set. All *Susceptible Isolates* were then scrutinized *in silico* for phylogroup and the presence of the PmrB E123D and Y358N amino acid substitutions. All isolates bearing the E123D amino acid substitution in this data set (*Susceptible Isolates*) belonged to phylogroup B2 (Fig. 2). Isolates from phylogroups B1 (*n* = 255) and C (*n* = 52) represented 95% of the 326 isolates demonstrating the Y358N amino acid substitution. All phylogroup C colistin susceptible isolates (*n* = 52) from this search demonstrated the Y358N amino acid substitution, and 97.6% of susceptible B2 isolates (*n* = 84) were found to have the E123D substitution.

To look more broadly at the distribution of the two PmrB variations of interest within *E. coli* phylogroups, 15,000 of 248,847 *E. coli* assemblies available in the NCBI Assembly database (31) were randomly selected and downloaded. No metadata was appraised for these isolates. To exclude fragmentary assemblies possibly lacking the motifs of interest, assemblies smaller than four megabases were removed, yielding 14,700 assemblies that were processed bioinformatically for phylogroup and the presence of E123D and Y358N amino acid substitutions (data set *NCBI Assemblies*). Again, all isolates demonstrating the PmrB E123D amino acid substitution (*n* = 2,376) belonged to phylogroup B2 (Fig. 2). Among isolates bearing the PmrB Y358N amino acid substitution (*n* = 7,795), most were phylogroup B1 isolates (68.6%, *n* = 5,347). In this data set, 98.2% of phylogroup B1 *E. coli* isolates bear the Y358N substitution, as do 98.1% of phylogroup C isolates and 83.7% of phylogroup E isolates.

**Fig 4:**
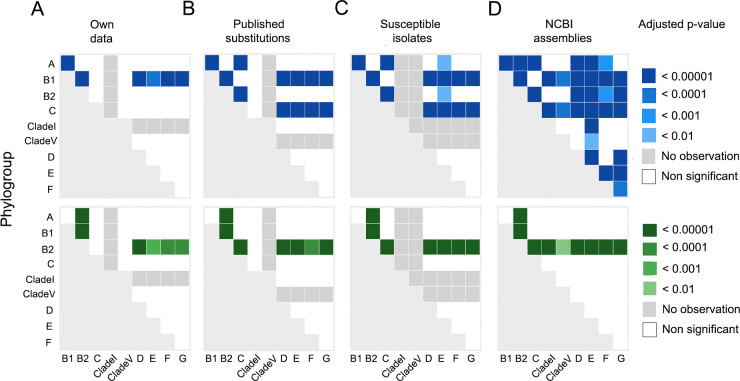
The *P*-values from pairwise Fisher’s Exact tests (Benjamini-Hochberg adjusted) comparing the proportion of isolates in each phylogroup demonstrating the PmrB Y358N amino acid substitution (blue) and PmrB E123D amino acid substitution (green) in the (A) *Own Data*, (B) *Published Substitutions*, (C) *Susceptible Isolates*, and (D) *NCBI Assemblies* data sets.

Within all data sets, there was a significant difference in the distribution of both PmrB Y358N (Fisher’s Exact test *P* < 0.001) and E123D (*P* < 0.001) amino acid substitutions among the phylogroups. Pairwise comparisons of the proportion of isolates demonstrating the Y358N variation within each phylogroup revealed significant differences between phylogroup B1 and phylogroups A, B2, D, E, F, and G (*P* < 0.001, Fig. 4) in all data sets. There were significant differences found between phylogroup B2 and phylogroups A, B1, D, E, F, and G in the proportion of isolates bearing the E123D substitution (*P* < 0.01, Fig. 4). In the *Published Substitutions*, *Susceptible Isolates*, and *NCBI Assemblies* data sets, the same statistically significant difference between phylogroups were observed. However, in these three datasets, there were also significant differences in the proportion of isolates demonstrating the PmrB Y358N amino acid variation between phylogroup C and phylogroups A, B2, D, E, F, and G (*P* < 0.001) and in the proportion of isolates demonstrating the PmrB E123D amino acid variation between phylogroups B2 and C (*P* < 0.001). Similar patterns were noted when the resistant and susceptible isolates within the *Published Substitutions* data set were considered separately (data not shown). However, no susceptible isolates in this data set belonged to phylogroup F. In the *NCBI Assemblies* data set, additional significant differences were also detected (Fig. 4).

**Fig 5:**
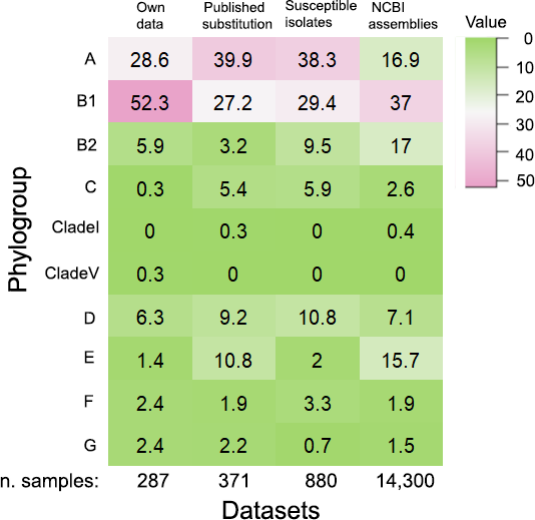
Phylogroup distribution of *E. coli* isolates within the four data sets scrutinized. Cell values are percentages of total isolates within each data set belonging to each phylogroup (column-wise).

The phylogroup with the largest variation in the proportion of PmrB substitutions between data sets was phylogroup E, with 2.5% of isolates in the phylogroup bearing the Y358N substitution at a minimum (*Published Substitutions*) but a maximum of 83.7% of phylogroup E isolates in the *NCBI Assemblies* data set. To examine if this observed dissimilarity resulted from differing phylogroup proportions in the data sets, the phylogroup composition of each data set was determined and then compared (Fig. 5). Phylogroup B1 was the most common phylogroup of isolates in the *NCBI Assemblies* (37.0%) and the *Own Data* (52.3%) data sets. In contrast, within the *Susceptible Isolates* and *Published Substitutions* data set, phylogroup A isolates were most common (38.3% and 39.9%, respectively), followed by isolates from phylogroup B1 (29.4% and 27.2%). Phylogroup E isolates were relatively common in the *NCBI Assemblies* and *Published Substitutions* data sets (15.7% and 10.8%) but were less frequent in the *Own Data* and *Susceptible Isolates* data sets (1.4%, 2.0%). Among phylogroup E isolates in the *NCBI Assemblies* dataset, 1,805 (78.1%) were found to belong to sequence type 11 (ST11) or ST335, of which 99.8% demonstrate the PmrB Y358N amino acid variation. *E. coli* isolates of the serotype O157:H7, 96.9% of which were classified as ST11, represented 12.0% of all genomes in the *NCBI Assemblies* data set. Twelve of 13 O55:H7 isolates (92.3%), identified previously as a recent precursor to O157:H7 (32), were assigned to ST335 in this data set. The other three datasets (*Own Data*, *Published Substitutions*, and *Susceptible Isolates*) did not contain any O157:H7, O55:H7, ST11, or ST335 isolates.

**Fig 6:**
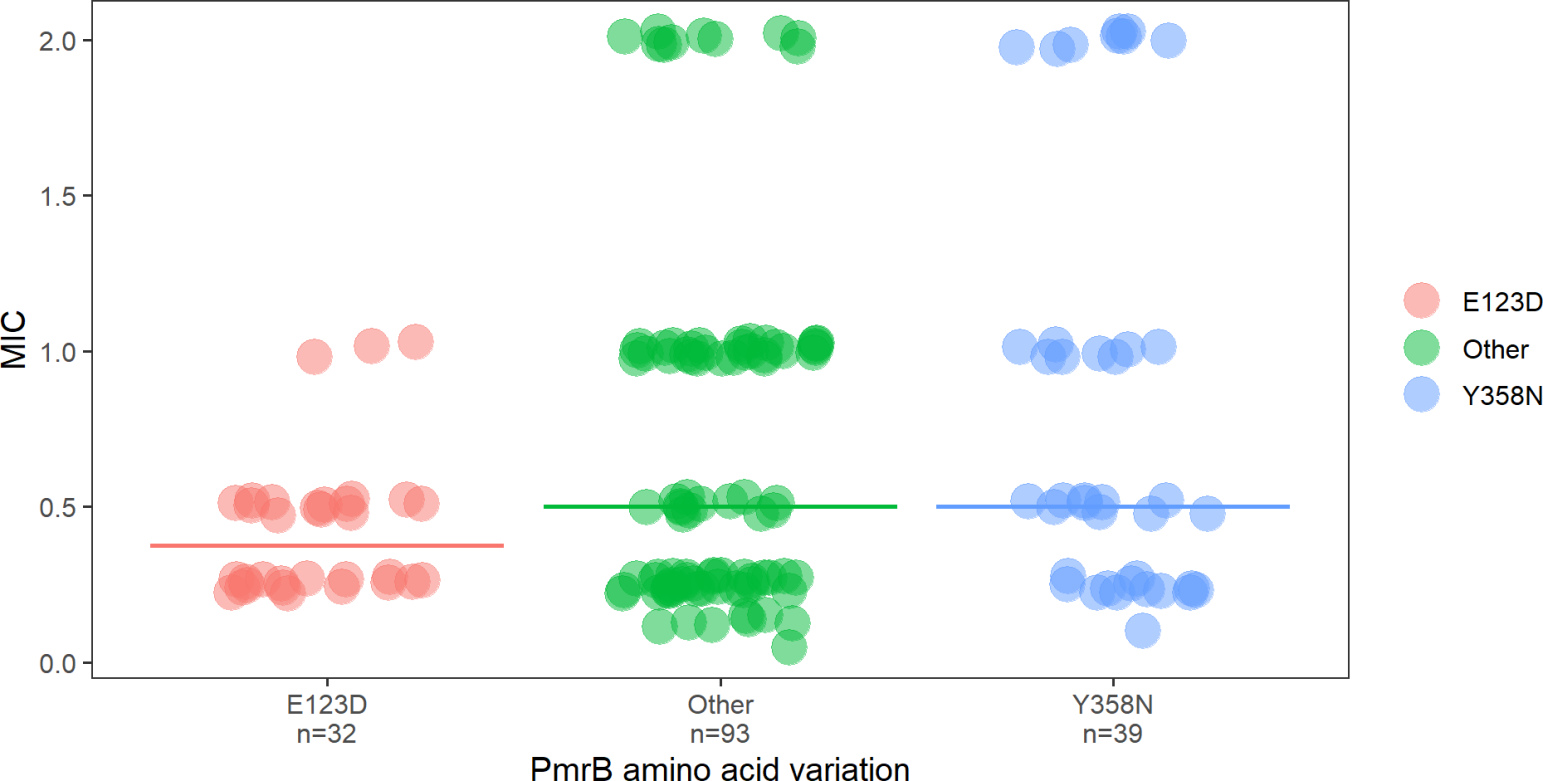
MICs as determined by broth microdilution of colistin susceptible *E. coli* isolates reported in the literature (*n* = 164) demonstrating the E123D, Y358N, or neither the E123D nor Y358N variations (“Other”). Horizontal lines indicate median MIC values for isolates bearing each amino acid variation.

To examine if the PmrB E123D and/or Y358N amino acid variations result in higher colistin MIC values even if the clinical breakpoint for colistin resistance was not surpassed, isolates with reported absolute values of colistin MIC by broth dilution were extracted from the *Susceptible Isolates* data set of published, publicly available genomes. One hundred sixty-four *E. coli* isolates met these criteria. There was no difference in the average mean ranks of MIC values between isolates demonstrating either PmrB Y358N, E123D, or neither amino acid substitutions (Kruskal-Wallis test H = 10.139, df = 5, *P* = 0.071; Fig. 6). In post hoc pairwise examinations, there were no significant adjusted pairwise differences in mean average ranks of MIC values between isolates bearing the Y358N amino acid substitutions or those that did not demonstrate either substitution of interest (Dunn test z = 0.835, *P* = 0.40). Comparisons between E123D-positive isolates and either Y358N-positive isolates or those with neither substitution neared statistical significance (z = 1.95, *P* = 0.078 and z = 2.35, *P* = 0.056, respectively); however, isolates with the E123D amino acid substitution demonstrated a lower median MIC.

## DISCUSSION

Two PmrB amino acid variations previously linked to colistin resistance in *E. coli*, E123D, and Y358N, appear closely associated with phylogeny. The E123D amino acid substitution was only found in *E. coli* isolates belonging to phylogroup B2, whereas the Y358N substitution is over-represented among B1 and C phylogroups and in some sequence types (ST11/ST335) of phylogroup E. Both substitutions were found in numerous colistin-susceptible isolates in addition to colistin-resistant *E. coli*.

The associations of the two amino acid variations with phylogeny were first noted in a large data set of uniquely sequenced, generic *E. coli* isolates that did not demonstrate colistin resistance on agar screening. These associations with phylogeny were similarly found in published resistant isolates with reported Y358N and E123D PmrB substitutions. However, additional isolates from those studies that were not assessed for the substitutions also demonstrated the substitutions with similar associations with phylogroup, and many of these isolates were phenotypically susceptible to colistin. This finding of PmrB Y358N and E123D in many susceptible isolates within the *Own Data* and *Published Substitutions* data sets called into question the relationship of the substitutions with colistin resistance. Thus, the literature was systematically searched for additional colistin-susceptible isolates with publicly available whole-genome information. The associations between PmrB substitutions of interest and phylogroup were corroborated in these susceptible isolates, with the Y358N variation distributing into phylogroups B1/C and the E123D variation found within phylogroup B2 isolates in similar proportions as the previous two data sets. Finally, if the associations between PmrB E123D and Y358N variations with phylogeny are valid, it would be expected that those relationships would be seen in all *E. coli* isolates, regardless of colistin resistance. Therefore, a large random sampling of publicly available *E. coli* assemblies was scrutinized. The strong associations between PmrB E123D and phylogroup B2, as well as PmrB Y358N with phylogroups B1 and C, were again observed, although a high proportion of phylogroup E isolates belonging to ST 11 and ST 335 also bore the Y358N variation. These patterns were statistically significant, except for the association between phylogroup C isolates and the PmrB Y358N variation within the *Own Data* data set, which lacked power with only four sampled isolates from that phylogroup.

In total, within the *Own Data*, *Published Substitutions*, and *Susceptible Isolates* data sets, more than 600 phenotypically colistin-susceptible *E. coli* isolates were found to demonstrate either the Y358N or E123D amino acid variation. The colistin MIC values among colistin-susceptible isolates pulled from the literature were not higher in isolates with the PmrB Y358N or E123D variations compared to isolates without either substitution. Therefore, it seems unlikely that the two substitutions alone confer phenotypic colistin resistance in *E. coli*.

The associations between phylogeny and PmrB E123D/Y358N amino acid variations are congruent with the phylogenetic history of *E. coli* as it is currently understood. The PmrB E123D amino acid variation was found only in B2 isolates, a phylogroup believed to branch basally within the *E. coli* phylogenetic tree (33, 34). At the more distant branches of the proposed evolutionary tree, phylogroups B1, A, and C are considered closely related “sister groups” (33–35). Additionally, Tantoso et al. (36) identified a subset of B1 isolates based on accessory gene content (including carriage of Shiga-toxin genes) that cluster not with phylogroups A and C but instead with phylogroup E. Combined, these phylogenetic relationships are consistent with this study’s finding that the PmrB Y358N amino acid variation is most common in phylogroup B1 and C isolates and Shiga-toxin producing ST11 isolates belonging to phylogroup E but is found much less frequently in other phylogroups.

There was a notably higher proportion of PmrB Y358N amino acid variations in phylogroup E isolates within the *NCBI Assemblies* database compared to the other three datasets. This higher proportion likely reflects a bias in the sampling of isolates submitted to the public database. Phylogroup E is a diverse group generally isolated less frequently than *E. coli* from phylogroups A, B1, B2, and D (37). However, included within phylogroup E isolates are many pathotypes exhibiting virulence factors of enterohaemorrhagic *E. coli* (EHEC), enteropathogenic *E. coli* (EPEC), enterotoxigenic *E. coli* (ETEC), and enteroinvasive *E. coli* (EIEC) (37) (6). These pathotypes may result in an over-representation of the phylogroup among isolates submitted to public sequence databases. The EHEC serotype O157:H7 belonging to ST11 dominates one of two clusters within phylogroup E identified in a recent extensive Mash-based analysis (35). It is of interest to researchers, clinicians, and epidemiologists due to its ability to cause outbreaks of severe gastrointestinal disease and hemolytic uremic syndrome in humans (38). Some countries maintain enhanced surveillance systems for O157:H7 (39), and whole-genome sequencing used to trace outbreaks related to the serotype could result in a disproportionately high number of these isolates with publicly accessible genomic information. The overrepresentation of O157:H7 isolates among *E. coli* isolates submitted to the EnteroBase database has been documented previously (40).

However, the EHEC, EPEC, EIEC, and ETEC pathotypes are only a small portion of the lineages within phylogroup E (37). In the data sets presented in this study, O157:H7 isolates belonging to ST11 and O55:H7 isolates belonging to ST335 were found to dominate the phylogroup E isolates pulled at random from the NCBI database (*NCBI Assemblies*) and carried the Y358N amino acid substitution at a very high rate. In the other three datasets (*Own Data*, *Published Substitutions*, and *Susceptible Isolates*), no isolates belonging to ST11 or ST335 were found. The proportion of phylogroup E isolates demonstrating the Y358N substitution was much lower in those three data sets.

In this examination, not all O157:H7 isolates were assigned to ST11 nor were all O55:H7 isolates assigned to ST335, as would be expected (6, 41). This study did not explore this further; however, this could result from errors in base calling or assembly affecting the *in silico* assignment of sequence types. In the *NCBI Assemblies* data set, *E. coli* assemblies were randomly selected from all publicly available in the NCBI database without consideration for assembly quality beyond the exclusion of very short assemblies (less than 4 MB).

To our knowledge, Brennan et al. (42) and Luo et al. (12) were the first to report the Y358N and E123D substitutions in PmrB. Brennan et al. did not detail how the significance of the amino acid variation was ascribed beyond the identification of “non-synonymous amino acid substitutions in genes previously shown to be associated with colistin resistance.” Luo et al. identified the substitutions through a comparison of *E. coli* isolates demonstrating high levels of colistin resistance (MIC ≥ 32 mg/L) with the lab strain *E. coli* K-12 MG1655 (a phylogroup A isolate that bears neither Y358N nor E123D) and nine colistin susceptible *E. coli* isolates. The genomes of the susceptible isolates compared in Luo et al. were not publicly available, so it was not possible to verify the phylogroup designations of the susceptible control strains used. Although the Y358N and E123D substitutions were also reported in numerous other studies (13–16, 43–45), additional comparisons of phenotypically colistin-resistant isolates with colistin-susceptible isolates to confirm the different proportions bearing the E123D and Y358N amino acid substitutions do not appear to have occurred. The presence of these variations in colistin-susceptible *E. coli* isolates was noted in two studies (20, 21) but only in a very small number of isolates (*n* = 3). In one of these studies (20), two colistin-resistant isolates also demonstrated the PmrB Y358N variation but were *mcr-*positive, and the authors stated that the Y358N substitution could not be considered a factor in the observed colistin resistance. Within the *Published Substitutions* data set in this study, only two publications (26, 30) also sequenced susceptible *E. coli* isolates. They did not comment on the presence of either Y358N or E123D in the non-resistant isolates; however, these susceptible isolates from the *Published Substitutions* data set were found to bear Y358N and E123D amino acid substitutions in similar proportions as resistant isolates in the same data set. The remaining studies in that data set did not have publicly available colistin-susceptible genomes.

The lack of close association between some amino acid variations in PmrA/PmrB and colistin resistance has been described in other studies. Wang et al. (46) identified several amino acid substitutions in PmrA and PmrB but noted that only the R81G substitution in PmrA could be associated with colistin resistance due to overexpression of PmrHGIJKLM. Although Quesada et al. (8) concluded that amino acid substitutions they identified in PmrA or PmrB proteins in *E. coli* isolated from poultry and swine were associated with polymyxin resistance, the two amino acid substitutions they delineated were R81S in PmrA and V161G in PmrB. They also identified D238G and Y357N point mutations in their isolates. However, these mutations were only found in colistin-susceptible isolates. It is not known if the Y357N mutation may be equivalent to the Y358N mutation noted herein.

Taken as a whole, these data indicate that the previously identified association between phenotypic colistin resistance and the PmrB Y358N and E123D amino acid variations *in E. coli* might be spurious. Two variables might appear to be correlated even in the absence of a causal relationship if the association examined is inappropriate or incomplete. In the case of PmrB E123D and Y358N substitutions, the initial comparison was not inappropriate, but rather the small number of colistin susceptible isolates (nine plus the lab strain K-12) selected without apparent consideration of phylogroup could have led to the identification of PmrB variations between colistin-resistant and -susceptible isolates that are unrelated to colistin susceptibility. Many subsequent studies identified the E123D and Y358N variations but only examined colistin-resistant isolates for the substitutions. Finding PmrB E123D and Y358N variations in these resistant isolates seemed to confirm the spurious association between the variations and phenotypic resistance. To our knowledge, complete epidemiological assessments of the relationship between PmrB Y358N or E123D and colistin resistance have yet to be performed to confirm the proposed association, despite its relatively common acceptance. This acceptance of PmrB E123D and Y358N variations as likely mechanisms of colistin resistance in *E. coli* emphasizes the importance of study design considerations when ascribing potential causation between chromosomal variations and a given phenotype, whether antimicrobial resistance or another phenotype.

Although they were among the first to identify the PmrB Y358N and E123D substitutions, Luo et al. (12) did acknowledge that yet unidentified factors could be involved in colistin resistance in *E. coli*. Two colistin-resistant isolates examined in their study did not exhibit any amino acid variations in PmrAB or other protein complexes known to be involved in LPS modification. Therefore, the identification of an amino acid substitution in PmrB should not be considered sufficient to confer colistin resistance in *E. coli* without further confirmatory evidence, as suggested by the results of this study. Binsker et al. (4) also urged caution in applying the results of genotypic analysis alone to the assignment of putative chromosomally encoded colistin resistance mechanisms. They stressed that genotypic studies should be complemented by experimental functional studies and supported by epidemiological data from animal or human surveillance programs. Another study (47) demonstrated that the 3′-UTR might be crucial in mediating colistin resistance caused by a single amino acid substitution in PmrB (G361A) and suggested possible roles of sRNA or RNA chaperones in mediation of colistin resistance. They encouraged further research in this area, supported by the warning of others that the mechanisms mediating colistin resistance in *E. coli* are not yet fully understood (48). Additional research assessing functional gene expression resulting from individual PmrB amino acid substitutions, and the effect of any noted gene expression differences on colistin MICs, could contribute to the understanding of mechanisms of colistin resistance in *mcr*-negative *E. coli* (49). Recombinant techniques could augment such examinations (49).

Several limitations to this study are acknowledged. There was no evidence of increased colistin MIC values in isolates with the Y358N or E123D amino acid substitutions. However, only a relatively small number of colistin-susceptible isolates with reported absolute MIC values determined by broth dilution (*n* = 164), the only method currently accepted by both the CLSI and EUCAST, were available in the literature. The possibility of “MIC creep,” incremental increases in MIC values due to the additive effects of two or more individual mutations, cannot be entirely excluded based on this examination. Additionally, evaluation of the potential additive effect of E123D or Y358N PmrB substitutions with other determinants of colistin resistance, resulting in elevated MIC values among colistin-resistant *E. coli* isolates, was beyond the scope of the current study. This possibility could bear future examination. Repurposing data from samples collected in multiple other studies, each with its sampling framework, and summarizing multiple studies retrospectively can introduce potential sampling bias unique to each study included. Although this may be present, examining broad patterns across many studies and multiple differing data sets attempted to minimize this concern. The *NCBI Assemblies* data set was very large, resulting in multiple statistically significant differences in PmrB proportions between phylogroups that might not be of practical importance in all instances. This consideration must temper the interpretation of the statistical relationships within that data set.

In conclusion, this study reinforces the importance of a thorough epidemiological and phylogenetic examination of *E. coli* isolates when identifying genotypic differences to explain observed phenotypic traits such as antimicrobial resistance. Given the complex phylogeny of the organism, the potential for spurious associations in such examinations may need to be considered.

## MATERIALS AND METHODS

### Sampling of newly sequenced isolates (*Own Data*)

All samples were collected in 2018 or 2019 in Alberta, Canada, and were derived from routine surveillance of broiler chicken and feedlot cattle [Canadian Integrated Program for Antimicrobial Resistance Surveillance (CIPARS)], retail beef and chicken meat (CIPARS), post-treatment wastewater (Advancing Canadian Water Assets), and private well water testing (Alberta Precision Laboratories).

CIPARS sampling protocols have been described in detail elsewhere (50). CIPARS performs yearly antimicrobial resistance surveillance of feedlot cattle and broiler chickens on farms selected to represent the national profile of farm sizes and locations in the respective food production commodities. Farms participate voluntarily. Briefly, pooled fecal samples were collected from feedlot cattle close to market weight and broiler chicken flocks during the last week of growth (chickens more than 30 days of age). For broiler chickens, randomly selected barns and floors (if multiple levels were present per pen or barn) were divided into quadrants, and one pooled sample with at least ten fecal droppings was collected from each quadrant (four pooled samples total). From each sampled feedlot one pooled fecal sample was collected from each of 10 pens (10 pooled samples total). Samples were shipped to the laboratory on ice.

For a sampling of retail meats, skin-on chicken legs, or wings and regular, medium, lean, and very lean ground beef were sampled from four stores per sampling week (typically one independent market or butcher shop and three chain stores) according to a stratified random sampling method guided by Statistics Canada census data weighted by province/region. Samples were placed in sealed zipper-type bags and shipped on ice.

Wastewater samples were collected weekly from 5 January 2018 to 8 November 2019, from three wastewater treatment plants in Calgary, Alberta, Canada, after final UV treatment. An overview of the wastewater treatment plants and collection methods is available elsewhere (51).

Alberta Precision Laboratories, an ISO 17025 accredited laboratory, performs testing of voluntarily submitted, private drinking water samples from wells in Alberta. Private citizens pick up sterile 250 mL sampling bottle packages from public health clinics throughout the province, which contain detailed instructions for sampling (52). Samples are to be collected from the residence’s cold-water supply line before any water treatment. Water samples are to be placed on ice and submitted immediately, as water was not tested if collected more than 24 h prior to lab submission. Well water samples in this study were submitted to the provincial laboratory from 19 July 2018 to 19 December 2019.

### Bacterial isolation and identification of newly sequenced isolates (*Own Data*)

Most retail meat and fecal samples were processed at the National Microbiology Laboratory (NML) in Saint-Hyacinthe, Quebec, although a portion of the primary isolation of fecal *E. coli* isolates was performed at the Agri-Food Laboratory of Alberta Agriculture and Rural Development. Thorough methods have been detailed elsewhere (50).

Briefly, 25 g of each pooled fecal sample (feedlot, broiler chicken) was combined with 225 mL of buffered peptone water (BPW), then one drop of the resultant mixture was streaked onto MacConkey agar and incubated for 18–24 h at 35°C ± 1°C. Suspect *E. coli* colonies (pink) were plated onto Luria-Bertani agar and incubated for 18–24 h at 35°C ± 1°C before identity confirmation with Simmons citrate and indole tests. Isolates with negatively indole tests had identity confirmed with a bacterial identification test kit (API 20E System).

For retail meat samples, 225 mL of BPW was mixed with one chicken leg or wing or 25 g of ground beef. Fifty milliliters of double-strength EC Broth were added to 50 mL of retained peptone rinse and incubated at 42°C ± 1°C for 24 h. One loopful of the incubated peptone rinse/broth mixture was streaked onto Eosin Methylene Blue agar and after 24 h incubation at 35°C ± 1°C, suspect colonies (metallic green) were plated onto trypticase soy agar (TSA) with 5% sheep blood. After incubation at 35°C ± 1°C for 18–24 h, isolates were screened for identity as described for fecal samples above.

Wastewater samples were filtered through a 0.45 µm Buchner filter, which was then placed on m-FC agar and incubated overnight at 44.5°C. Blue colonies (consistent with coliforms) were sterilely picked and individually added to 100 µL of sterile Luria-Bertani (LB) broth in a 96-well plate. The well plates were incubated at 37°C overnight, and then sterile LB broth/glycerol was added to a final concentration of 25% glycerol for cryopreservation at −80°C.

One random post-UV treatment isolate that demonstrated blue or dark blue colony morphology on m-FC agar was randomly selected from each available wastewater sampling date at each treatment plant, yielding 192 samples. Initial screening on X-gluc agar identified suspect *E. coli* (blue colonies), of which a single well-isolated colony was picked and plated onto TSA agar with 5% sheep’s blood and then incubated at 37°C for 18–24 h. Bacterial identification was confirmed with the API 20E Identification System following the manufacturer’s specifications (bioMerieux, Marcy-L’Etoile, France). The Water Quality Services Laboratory (UEP—Water Resources, City of Calgary) performed the filtration and plating of water samples, whereas selection from selective media, cryopreservation, and further processing of isolates occurred at the University of Calgary.

At Alberta Precision Laboratories, 88 presumptive *E. coli*-positive (yellow, fluorescence-positive) well water samples were identified with the Colilert enzyme-substrate system (Idexx Laboratories, Westbrook, ME, USA) following the manufacturer’s protocol, then individually cryopreserved at −80°C with or without 15% glycerol before transfer to the University of Calgary. Each sample was thawed on ice and 1 mL was added to 9 mL of sterile tryptic soy broth. After 18–24 h incubation at 37°C, 10 µL of each sample was streaked on X-Gluc agar and incubated for 18–24 h at the same temperature. A random blue isolate from each sample was selected, plated onto TSA agar with 5% sheep’s blood, and incubated at 37°C for 18–24 h. Identity was then confirmed using the API 20E Identification System. All *E. coli* thus isolated were cryopreserved at −80°C in 15% glycerol.

### Antimicrobial susceptibility testing of new sequences isolates (*Own Data*)

Antimicrobial susceptibility testing (AST) was performed by automated broth microdilution (Sensititre Trek Diagnostic Systems Ltd., West Sussex, England) using the National Antimicrobial Resistance Monitoring System (NARMS) Gram-negative CMV4AGNF AST plates. The antimicrobials tested included amoxicillin-clavulanic acid, ampicillin, azithromycin, cefoxitin, ceftriaxone, chloramphenicol, ciprofloxacin, gentamicin, meropenem, nalidixic acid, streptomycin, sulfisoxazole, tetracycline, and trimethoprim-sulfamethoxazole. Results were interpreted according to CLSI M100-S26 (53) except for azithromycin and streptomycin, for which CLSI interpretive criteria for Enterobacteriaceae were not available. Breakpoints from NARMS were used for those antimicrobials. AST of retail meat and fecal sample-derived isolates was performed at the NML; wastewater and well water AST was completed at the University of Calgary.

All isolates were screened for growth on Mueller-Hinton agar containing 2 mg/L colistin sulfate. Testing of fecal and retail meat isolates occurred at the National Microbiology Laboratory as described previously (54), while water isolates were screened at the University of Calgary following the same procedure except for the control strains used (negative controls*—Pseudomonas aeruginosa* ATCC-27853 in addition to *E. coli* ATCC-25922 and positive control *E. coli* ATCC-BAA-3170).

### DNA extraction and whole genome sequencing of newly sequenced isolates (*Own Data*)

A stratified random sampling framework was used to select 288 isolates for whole genome sequencing, with strata defined by source and class-level phenotypic antimicrobial resistance. Random sampling within each stratum employed the sample_n() function in the base module of RStudio with a seed set using a random number generator. Using this methodology, 48 samples were selected from each source: 16 pan-susceptible isolates, 16 isolates resistant to one or two classes of antimicrobials, and 16 isolates demonstrating resistance to three or more classes of antimicrobials. For well water, there was an insufficient number of isolates exhibiting phenotypic resistance to meet the sampling criteria. Therefore, all phenotypically resistant well water isolates were selected for sequencing (*n* = 4), with the remaining 44 isolates from that source selected randomly from pansusceptible samples.

DNA extraction utilized the DNeasy Blood and Tissue Kit (Qiagen, Toronto, ON, Canada) and followed the manufacturer’s protocol for Gram-negative bacteria. DNA was quantified using a Qubit Fluorometer 3.0 (Thermo Fisher Scientific, Mississauga, ON, Canada), and sample purity was assessed by spectrophotometry (NanoVue Plus; GE HealthCare, Chicago, Illinois). Whole-genome sequencing was performed at the Centre for Health Genomics and Informatics at the University of Calgary (Calgary, AB, Canada) using either the RIPTIDE High-Throughput Rapid Library Prep (HT-RLP) (iGenomX/Twist Bioscience, Carlsbad, CA, USA) with sequencing on the Illumina NovaSeq 6000 platform (San Diego, CA, USA) or the NEBNext Ultra II library prep (New England BioLabs Inc., Ipswich, MA, USA) with sequencing on the Illumina MiSeq (v2, San Diego, CA, USA). Both protocols generated 2 × 250 base pair paired-end reads.

### Bioinformatic analysis

After quality assessment of resultant reads with fastqc v 0.11.9 (55), the reads were trimmed with fastq-mcf v 1.04.807 (56, 57) using a skew percentage of 10 (-k 10), minimum length of 120 (-l 120), a sliding window of three (-w 3), and a minimum phred quality score of 20 (-q 20). The reads were then assembled with Shovill v 1.0.9 (https://github.com/tseemann/shovill) using the SPAdes backbone with a quality assessment performed with Quast v 5.2.0 (58). Phylogroup assignment was performed *in silico* using ClermonTyping v 21.03 (59) and antimicrobial resistance was predicted *in silico* with AMRFinderPlus v 3.10.45 (19) using the default settings and—organism Escherichia flag. In cases of disagreement between the ascribed phylogroup and Mash group (e.g., ClermonTyping note: "Phylogroup doesn't match the mash closest neighbor’s group! This could indicate a mutation affecting the binding of a primer”), the Mash group designation was used. Pathotype prediction and MLST were conducted using ECTyper (60) and MLST (https://github.com/tseemann/mlst), respectively. MLST makes use of the PubMLST website (https://pubmlst.org/) developed by Keith Jolley (61) and is sited at the University of Oxford. The development of that website was funded by the Wellcome Trust.

### Literature search for published isolates

A literature search of the Web of Science (Clarivate *Web of Science*, 2023) and PubMed (62) databases was performed on 23 August 2023, with the following search parameters: Colistin AND (“*E. coli*” OR “Escherichia coli”) (Topic) and (suscept* OR resistant*) (Topic) and sequencing (Topic).

The search was limited to articles published from 2017 to the present. This search yielded 961 studies, which were parsed by hand to identify (i) studies that reported one or more *E. coli* isolates bearing either the Y358N or E123D amino acid substitutions and had publicly available genomes, and (ii) studies that reported *E. coli* isolates with publicly available genomes that were non-resistant to colistin by broth dilution, the only AST method approved by both CLSI and EUCAST for colistin susceptibility testing. Preprints were excluded, as were studies in which the isolates or publicly available genomes could not be matched to colistin susceptibility results. All isolates were selected for inclusion before determination of phylogroup or PmrB amino acid sequence, but isolates were excluded if they were found on subsequent analysis to be non-Escherichia or if the publicly available assemblies were identified as contaminated by the NCBI database. No sample size calculation was performed, as all available isolates that met the criteria were included. Isolates from a given study could be included in more than one dataset if the criteria for each were met.

The data set of colistin-susceptible isolates was further parsed for isolates with published, absolute colistin MIC values. Isolates were only included if all isolates in the study had absolute MIC values to avoid biased exclusion of only those isolates with the lowest MICs (e.g., prevent exclusion of all isolates with a MIC < 0.5 mg/L while retaining isolates with MICs of 1 or 2 mg/L).

### Bioinformatic processing of published isolates

All publicly available Sequence Reads Archive submissions that met the selection criteria were trimmed and assembled as described above (short-reads) or assembled with Flye v 2.9.2-b1886 (long-reads) (63). As the Y358N and E123D PmrB substitutions were first identified through comparison to *E. coli* K-12 substrain MG1655 (12), an assembly for K-12 MG1655 was downloaded from the NCBI database (GenBank Accession GCA_000005845.2). All downloaded genomes were screened for the Y358N and E123D PmrB amino acid substitutions with AMRFinderPlus and assigned to phylogroups with ClermonTyping in the same manner as above. Pathotype prediction and sequence types were determined *in silico* as described above.

### Random sampling of published assemblies

A random sample of 15,000 publicly available *E. coli* assemblies was analyzed to determine the prevalence of PmrB E123D and PmrB Y358N substitutions in isolates not selected for colistin susceptibility. Accession numbers of all available *E. coli* assemblies were retrieved from the NCBI Assembly database using the R package Rentrez (64). These assembled sequences were downloaded to a local server utilizing custom Python and Bash scripts. Only FASTA files with assembled sequences exceeding four megabytes were considered for further analysis to ensure the exclusion of incomplete assemblies. Phylogroups, PmrB genotypes, pathotypes, and sequence types were determined using ClermonTyping, AMRFinderPlus, ECTyper, and MLST, respectively, as for the other isolates in the study. The resulting data were processed with Bash scripts. Random samples of genomes, ranging from 100 to 14,700 (total *n* = 147), were drawn without replacement and without exclusion of isolates included in other data sets within this study using a custom Python script. Lastly, the data were summarized and visualized using in-house R scripts. The bioinformatics pipeline and scripts used are publicly available at (https://github.com/jjovelc/EcoliGenomics/tree/main/PmrB_susbtitutions).

The initial sample size for the *NCBI Assemblies* data set was an estimate, then assessed by graphing the proportions of each phylogroup demonstrating each PmrB amino acid substitution (Y358N/E123D/other) as 100 genomes were added sequentially (Fig. S1). When stabilization in the proportions was reached despite the addition of further assemblies, the sample size was assumed sufficient.

### Statistics

Results were collated in Excel (65) and then analyzed using RStudio (66, 67). Differences in proportions of each PmrB amino acid substitution (Y358N, E123D) were analyzed with Fisher’s Exact Test due to numerous small cells in expected values [fisher.test() function in the stats package]. Post hoc pairwise comparisons with Benjamini-Hochberg adjustment for false discovery rate were performed using the pairwise_fisher_test() function in the rstatix package (68). A Kruskal-Wallis test [kruskal.test() function in the stats package] was used to assess differences in mean ranks of absolute MIC values in the *Susceptible Isolates* data set, comparing isolates bearing the Y358N substitution, isolates carrying the E123D substitution, and isolates without either amino acid substitution. Post hoc pairwise comparisons were performed using Dunn’s test with Benjamini-Hochberg adjustment for false discovery rate [dunn_test() function of the rstatix package].

## Data Availability

Nucleotide sequences of isolates in the *Own Data* data set have been submitted to the NCBI Sequence Read Archive (SRA) database under BioProject PRJNA1120594 with accession numbers provided in Table S1. Accession numbers for samples in the *Published Substitutions* and *Susceptible Isolates* data sets are also listed in Table S1. Assembly identifications for isolates in the *NCBI Assemblies* data set are available from the authors upon request.

## SUPPLEMENTAL MATERIAL

Figure S1 spectrum.00532-24-s0001.tif PmrB variations in different sample sizes of assemblies from the NCBI database.

Table S1 spectrum.00532-24-s0002.xlsx Accession numbers, colistin susceptibility, PmrB mutations, and phylogroup of isolates.

ASM does not own the copyrights to Supplemental Material that may be linked to, or accessed through, an article. The authors have granted ASM a non-exclusive, world-wide license to publish the Supplemental Material files. Please contact the corresponding author directly for reuse.
